# Correction: Trehalose supports the growth of *Aedes aegypti* cells and modifies gene expression and dengue virus type 2 replication

**DOI:** 10.1371/journal.ppat.1013644

**Published:** 2025-10-30

**Authors:** Andrew D. Marten, Douglas P. Haslitt, Chad A. Martin, Akshitha Karthikeyan, Daniel H. Swanson, Karishma Kalera, Ulysses G. Johnson, Benjamin M. Swarts, Michael J. Conway

The Data Availability statement for this article is incorrect. The correct statement is: FASTQ files for each sample are available in the NCBI SRA database (BioProject ID: PRJNA936836 and PRJNA882813). Minimal data sets for all figures are provided in Supplementary Table 4.

S4 Table is omitted from the list of Supporting Information. It can be viewed below.

## Supporting information

S4 Table Minimal data set for all graphs. (XLSX)
