# Supplementary material for: Trehalose supports the growth of Aedes aegypti cells and modifies gene expression and dengue virus type 2 replication
Source: PLoS Pathog. 2025 May 6;21(5):e1012795. doi: 10.1371/journal.ppat.1012795 (PMC12077775; doi:10.1371/journal.ppat.1012795)
Supplement: Attachment — Submitted filename: Response to Reviewer.docx [file ppat.1012795.s005.docx]

**Reviewers' Comments:**

Reviewer #1: Aspects that could be clarified or further improved from my perspective:

• Figure 1: A) Why is the accession number for TPP accompanied by a question mark? Please clarify. B) The legend could be more descriptive, providing clearer guidance on how to interpret this panel, for SignalP non-users.

Thank you. This section did need to be clarified after review. We have found that there is expression of TREH in Aag2 cells, but that the TPS (AAEL006446) gene that was automatically predicted from the *Ae. aegypti* genome sequence was absent in the analysis. TPP (AAEL010684) was predicted by UnitProtKB/TrEMBL and is reported in VectorBase but is low homology when compared with TPP from other species. We originally placed a question mark because we were skeptical that this was the real TPP, although we don’t have evidence to support that one way or the other. The question mark has been removed. We revised the language in the manuscript to reflect this. “An RNA-Seq dataset that characterized gene expression of Aag2 cells revealed high expression of trehalase (TREH; AAEL009658), but the previously predicted trehalose-6-phosphate synthase (TPS; AAEL006446) was absent in the analysis (42). The gene that provides trehalose 6-phosphate phosphatase (TPP; AAEL010684) activity in *Ae. aegypti* was predicted by UniProtKB/TrEMBL and had high expression in Aag2 cells (Figure 1A). Based on this information, it was unclear if Aag2 cells could synthesize trehalose, but they could likely metabolize trehalose.”

We added more information in the figure legend so that the reader can identify the exact location of the predicted signal peptide and understand that the plots identify the n-terminal, hydrophobic, and c-terminal regions of the signal peptide. The new language is as follows, “(C) SignalP 6.0 analysis of TREH amino acids sequence predicts a Sec/SPI signal peptide cleavage site at S30 and associated n-terminal (n), hydrophobic (h), and c-terminal (c) regions.”

• Lines 296–297: I was unable to locate TPS (AAEL006446) in Tables S1, S2, or S3 of the referenced paper (Reference 42). While I found “AAEL010684,” its associated function is listed as “probable histone-lysine N-methyltransferase Mes-4, transcript variant X2,” not TPP. Furthermore, according to Tables S1 and S2 of Reference 42, TPP corresponds to other accession numbers and not AAEL010684.

Thank you. This section did need to be clarified after review. We have found that there is expression of TREH in Aag2 cells, but that the TPS (AAEL006446) gene that was automatically predicted from the *Ae. aegypti* genome sequence was absent in the analysis. TPP (AAEL010684) was predicted by UnitProtKB/TrEMBL and is reported in VectorBase but is low homology when compared with TPP from other species. The annotations in the cited manuscript are imperfect, which is why we relied on the unique identifiers. We revised the language in the manuscript to reflect this. “An RNA-Seq dataset that characterized gene expression of Aag2 cells revealed high expression of trehalase (TREH; AAEL009658), but the previously predicted trehalose-6-phosphate synthase (TPS; AAEL006446) was absent in the analysis (42). The gene that provides trehalose 6-phosphate phosphatase (TPP; AAEL010684) activity in *Ae. aegypti* was predicted by UniProtKB/TrEMBL and had high expression in Aag2 cells (Figure 1A). Based on this information, it was unclear if Aag2 cells could synthesize trehalose, but they could likely metabolize trehalose.”

• Line 300: Including a screenshot from the PSORT Wolf analysis, similar to the presentation of SignalP 6.0 in Panel 1C, would greatly enhance clarity. Alternatively, this could be provided in the Supplementary Figures for additional reference.

The final output of this analysis is a simple line of text and doesn’t provide a graphical representation of the data. There is a very long table that identifies each neighbor that the protein is related to based on homology but I’m not sure if this makes the analysis easier to understand. We opted to convert this data into a pie chart and present that as Figure 1B. Our follow-up experiment that conclusively shows that there is trehalase activity in cell-free supernatant should be more helpful in solidifying that at least some TREH is secreted. Please see the PSORT Wolf output examples below.

• Line 302: A more conclusive experiment, such as isolating the supernatant from cells, adding it to a trehalose solution, and subsequently measuring the glucose content, would provide stronger experimental proof. Trehalase could be present within the cells, converting trehalose into glucose as a non-secreted protein, especially considering trehalose is reported to enter the cells via passive transport. Additionally, if antibodies are available, a western blot analysis could help clarify this point.

Thank you for identifying a key experiment that can reveal trehalase activity in cell-free supernatant. We were able to provide a new dataset using cell free supernatant from Aag2 cells that were grown in trehalose-containing cell culture medium to show conversion of trehalose to glucose over a 72-hour time course. Importantly, this activity was inhibited by 1 mM trehalase inhibitor validamycin A (ValA) and did not occur when trehalose-containing cell culture medium that had not contacted Aag2 cells was used. This new figure is included as Figure 1C and provides strong support that at least some TREH is secreted into the extracellular environment.

• Figure 2F: The presentation of this experiment is not clear to me. If I understand correctly, the overall goal is to compare the efficiency of trehalase inhibitors. ValA, an inhibitor of trehalase, was tested alongside analogues of trehalose. Could you clarify whether these analogues also act as inhibitors by competing with native trehalose? Additionally, is it known how ValA works?

Thank you. The goal was to show proof-of-principal that we can use this *in vitro* model to screen novel trehalose analogues that may or may not serve as trehalase inhibitors. Validamycin A (ValA) is a strong competitive inhibitor of the trehalase enzyme from a wide range of species including bacteria, fungi, and arthropods. ValA is structurally similar to trehalose, which is why it is able to occupy and prevent interaction between trehalase and trehalose. Other trehalose analogues have previously been identified that are able to inhibit trehalase with different efficiencies, which can also be species-specific. I revised the text so that ValA’s mechanism of action is described and so that the reader understands that other trehalose analogues can have inhibitory activity against trehalase.

“ValA is a competitive inhibitor of trehalase and other trehalose analogues have previously been shown to inhibit trehalase. To directly test the impact of trehalose analogues on trehalase activity, a dose-dependence assay was performed that quantified conversion of trehalose to glucose. ValA maintained ~75% activity as a trehalase inhibitor at the lowest concentration tested (i.e., 0.008 mM) (Figure 2F). Although 5-ThioTre and 6-TreNH2 maintained ~75% activity at the highest concentration tested (i.e., 1.0 mM), they had no activity at the two lowest concentrations (Figure 2F).”

• Line 342: In the RNAseq experiment, the upregulation and downregulation observed in the trehalose-containing medium are compared to which condition? Is it the glucose-containing medium?

That is correct. The section states that, “RNA-Seq was performed to characterize the differentially expressed genes in Aag2 cells maintained in glucose and trehalose-containing cell culture media.”
• Line 343: You mention that TREH expression did not change; however, I could not locate it in Supplementary Table 1. Is its absence from the table the reason for concluding that its expression did not change, or was it assessed elsewhere?

TREH (AAEL009658) is found on row 4440. The annotations that were provided with the analysis are imperfect. There was not a statistically significant difference between the two groups.

We confirmed this with RT-qPCR in Figure 3B. There was no change in TREH expression over the course of three days in the two types of cell culture media.

• Line 347: The statement regarding changes in genes involved in “the composition of the cell membrane [...] mitochondrial function” is unclear. It is not apparent where this information is extracted from, as it does not seem to be shown in Figures 3C or D. Could you provide a reference to the data supporting this statement?

Thank you. The mismatch was due to a previous DAVID Functional Annotation result that was not included in the figure. We aligned the language to the g:Cost Functional Profiling tool summary, which is shown in the pie charts in Figures 3C-D. The language in the text now states, “Functional annotations of differentially expressed genes also showed significant changes in genes involved in cell-substrate adhesion, integral/intrinsic membrane components, metabolic pathways, drug metabolism, transmembrane receptor protein kinase activity, and enzyme-linked receptor protein signaling (Figure 3C-D).” We also restated that the g:Cost tool was used in the figure legend.

• Line 348: A closing bracket appears to be missing.

Thank you. This has been corrected.

• Line 353: The sentence is unclear. If trehalose is already provided in the cell medium, it seems unnecessary for the cells to express TPP to obtain trehalose. Could you clarify what is being tested in this context? Are you investigating whether Aag2 cells are capable of producing trehalose themselves?

Thank you. We removed the reference to TPP since it’s homolog in *Ae. aegypti* has very low homology and there is no experimental evidence confirming that it has enzymatic activity that would promote trehalose synthesis. Our observation that Tret1 expression increases in the presence of trehalose is sufficient to posit that Aag2 cells may uptake trehalose from the extracellular environment. We changed the language of the text to the following, “Considering that Aag2 cells increase expression of Tret1 in response to trehalose, it is reasonable to expect that these cells can acquire trehalose from the extracellular environment (Supplementary Tables 1-2).”

• Line 366: The main conclusion of this section seems to be missing.

The conclusion was placed at the end of the second/last paragraph in the section. It states that, “these results support that the trehalose probe entered Aag2 cells through a process of facilitated diffusion, which is consistent with the function of known trehalose transporters such as Tret1 (43).”

• Line 451: If the goal is to assess autophagy, why were autophagy markers, such as Lysotracker, not tested on the cells with and without trehalose? Including such markers would strengthen the conclusions.

Thank you for the question. We were not interested in autophagy, mostly because we failed to identify any upregulated genes in the RNA-Seq dataset that were associated with autophagy. That was not a category that was significantly upregulated. In contrast, integral/intrinsic membrane components were significantly differentially expressed. We added to the text that, “Importantly, there were no autophagy-specific genes or pathways that were significantly upregulated in the RNA-Seq dataset.” We were also cautious regarding the testing of autophagy because previous research in this area has shown contradictory results in insect cells. We wrote that, “The role of autophagy in arbovirus infection in insect cells is less clear, and conventional autophagy inducers and inhibitors may respond differently in insect cells (44, 50).” This recent observation limits the use of many tools used for autophagy research in insect models and leads to a less than comprehensive investigation of autophagy. We knew that a proper investigation of autophagy in insect cells would require much more study including validation of the commercial tools in insect cells. Nile Red staining allowed us to quantify a broader biological change in these cells that was consistent with our RNA-Seq data without attributing it to a specific process.

• Line 454: Could you also test blocking autophagy in these cells when trehalose is provided? This would help determine whether the higher DENV levels are promoted by autophagy. For example, chloroquine could be used to inhibit autophagy.

This is theoretically possible, although as we now state in the paper, “Importantly, there were no autophagy-specific genes or pathways that were significantly upregulated in the RNA-Seq dataset.” While trehalose has been shown to induce autophagy in mammalian cells, the RNA-Seq data provided no evidence that is happening in Aag2 cells. We state in the paper that, “Trehalose can also be converted to glucose and used as a substrate for lipogenesis (42).” This was supported by our RNA-Seq data, which showed upregulation of many genes associated with lipogenesis. That is why we focused on Nile Red staining and quantifying intracellular lipid content.

• Line 483: This section appears to lack a clear conclusion.

Thank you. We have added a brief conclusion at the end of this section that states, “These data reveal that ValA toxicity is trehalose-dependent in Aag2 cells and that it simultaneously reduces cell viability, DENV shedding, and DENV infectivity.”

• Line 511: The statement “Aag2 cells were not derived from fat body cells or tissue” raises a question. Since these cells originate from embryos, wouldn’t it be expected that some could differentiate into fat body-like cells and potentially excrete Tret1?

Absolutely. Aag2 cells were once pluripotent and have likely developed into a heterogenous population of cells that don’t perfectly match any cell type in a living mosquito. The Tret1 that is expressed may or may not reflect the average peripheral cell/tissue, although it is important to note that Tret1 and TREH expression were not artificially selected because, up to this moment, laboratories have grown these cells in high glucose cell culture medium. Aag2 cells have never been exposed to trehalose or selected in a trehalose-rich environment, yet they are responsive to trehalose. The possibility that peripheral cells/tissues may uptake trehalose as a functional molecule rather than as glucose is an open and important research direction.

• Line 514: Which experiment could be performed to test this hypothesis? Would using Tret1 RNAi be a suitable approach?

Unfortunately, the specific *Ae. aegypti* Tret1 gene is unknown because there are at least a dozen homologues. We did identify some sugar transporters that were annotated as facilitated trehalose transporters that were upregulated in the presence of trehalose, but confirmation of the specific transporter that is mostly responsible for trehalose transport would likely require exogenous expression of a panel of Tret1-like genes in a cell types that does not uptake trehalose such as a mammalian cell line, and then RNAi could augment this approach. This would be a very large individual study comparable to the following to characterize a single gene or collection of genes:
Kikawada T, Saito A, Kanamori Y, Nakahara Y, Iwata K, Tanaka D, et al. Trehalose transporter 1, a facilitated and high-capacity trehalose transporter, allows exogenous trehalose uptake into cells. Proc Natl Acad Sci U S A. 2007;104(28):11585-90.

• Line 517: Could you speculate on why this effect is also observed, to some extent, when glucose is provided?

We added that, “Increasing the concentration of glucose was less able to prevent uptake of NBD-Tre suggesting that there may have been competition at the level of sugar-specific transporters.” We presume that, while there is specificity for substrates at these transporters, there is some similarity between glucose and trehalose (2 glucose molecules) so even high glucose levels and partially inhibit probe uptake.

• Line 566: How does the freeze-thaw event relate to virus replication in this context? Could you clarify its relevance?

Thank you. Yes, we revised the statement to the following, “Trehalose may provide some membrane or protein stabilizing activity that either allows virus production and shedding to occur more efficiently or it simply allows more virus to survive the freeze-thaw event that occurs after removing a virus stock from the -80°C freezer prior to experimentation.” We always place our virus stocks in a -80°C freezer, which then requires 1 freeze-thaw cycle before experimentation. If something is present that stabilizes the virus during this process that sample would likely be more infectious.

• Figure 9E, F: When considering the number of cells, is the level of virus per cell consistent between the glucose and trehalose conditions?

Yes, both groups were inoculated with the same amount of DENV and the same number of cells were inoculated on D0. We were able to see the same increase in FFU/well in the untreated trehalose-fed cells compared to the glucose-fed cells that we saw in Figure 6B. Over the course of the 5-day treatment, the number of viable cells decreased in the trehalose-fed cells that were exposed to ValA. We state in the discussion that, “1.0 mM ValA causes a 90% reduction in cell viability 5 days post-treatment in Aag2 cells that were maintained in trehalose-containing cell culture media. The reduction in cell viability correlated with an approximately 75% decrease in DENV vRNA shedding.” Therefore, the loss of cell viability correlated with the loss of DENV shedding and infectivity. The loss of DENV shedding and infectivity was clearly the result of the loss of cell viability and not an independent effect of ValA since ValA treatment had no effect on DENV shedding and infectivity in cells grown without trehalose.

• Line 582: You suggest that vertical transmission may not play a significant role. It is worth noting that a recent study by Julien Pompon demonstrated larval transmission through contamination with adult feces in Culex mosquitoes infected with West Nile virus. This highlights that such mechanisms could contribute to virus maintenance in the field.

Thank you. We agree that vertical transmission can occur under the right circumstances but suggest that it is not the main driver leading to maintenance of DENV in the environment. We state in the discussion, “that vertical transmission is less important for the maintenance and transmission of DENV in a natural environment.” It can occur and it has been documented to occur in laboratory settings at low levels, but its overall impact on driving human transmission and disease is low.

• Lines 586–588: The sentence is too long, and should be splitted into two sentences.

Thank you. That has been revised.

• Line 656: A period is missing after “RNA.”

Thank you. That has been revised.

• Statistical significance is missing or unclear across several figures. In Figure 2, the significance of the difference at 7 dpi in panel B is not indicated, and statistical analyses are missing for all panels except A. In Figure 3B, no statistical analyses are provided to support the results. In Figure 4C, statistical analysis bars are absent despite a decrease being observed in Panel E for the two lower glucose concentrations, as mentioned in the text. Including statistical significance throughout these figures would strengthen the clarity and robustness of the findings.

Thank you. We agree. Most of these figures had a non-significant result, so we added an “ns” designation to those results, and clarified in the text that these were non-significant or that there were no changes between groups. Please see examples of the updates below.

I found the manuscript to be well-organized, clearly written, and supported by robust data. While there are areas that could benefit from further clarification or enhancement, they do not significantly detract from the overall strength of the study. It is important to emphasize that I believe this manuscript does not require additional experimental data to be accepted for publication.

Thank you.

That said, the conclusions could be further strengthened by demonstrating in vivo in Aedes aegypti that ValA not only prevents mosquito flight but also reduces DENV levels and prevents its excretion in saliva. While not essential for this study, which focuses on developing an in vitro tool to test trehalase inhibitors, this could be an interesting direction for future research. I would be open to collaborating on such experiments if the authors are interested.

We agree that future studies should be supported to test the role of trehalose and its inhibitors on mosquito physiology and virus infection.

In addition, I would find it interesting to explore whether the presence of trehalose or its conversion into glucose via trehalase leads to an increase in lipids, which consequently supports DENV replication. Using ValA on cells to observe a potential decrease in lipid levels or blocking lipid production to assess its impact on DENV replication could provide valuable insights. Again, while not required, these experiments could further enhance the manuscript’s impact.

We agree that these are important studies to perform in the future.

Reviewer #2: The manuscript by Andrew D. Marten et al. investigates the effects of trehalose metabolism in mosquito cells. They found that trehalose promotes cell growth and enhances DENV infectivity. However, I have several concerns that need to be addressed:

-The authors claim that trehalose promotes cell proliferation. Since trehalose is metabolized into glucose, the mechanism by which trehalose promotes cell growth needs to be discussed in more detail. This explanation is essential to understand the underlying biological processes involved.

Thank you. Trehalose-containing cell culture media supports the growth of Aag2 cells, but it does not promote growth beyond glucose-containing cell culture media. In Figure 2B we showed that both lead to equivalent growth. It is important to note that the concentration of trehalose that was used was half of the glucose concentration, which leads to equimolar glucose concentration if all trehalose is converted to glucose.

-While the authors show that trehalose promotes virus infection, the mechanism behind this effect remains unclear. Given the transcriptome data, a more thorough investigation into how trehalose regulates virus infectivity is needed to clarify this relationship.

Thank you. In this manuscript, we were able to show that there is no co-localization between trehalose and DENV, so we do propose that the mechanism is related to broader changes to the transcriptome and likely metabolic pathways related to lipid metabolism. There is probably not a single gene that is responsible. Trehalose benefits DENV through a significant change in the transcriptome compared to cells fed with glucose media. That said, we were able to focus on the stage of the virus life cycle that was enhanced, which occurred after attachment and led to increased viral replication, shedding, and specific infectivity. This correlated with an increase in intracellular lipids, which was supported by the transcriptomic data. Future studies will need to characterize the lipid environment in these cells and the lipid composition of virions produced in cells fed with trehalose. We are excited to work on those studies.

-Line 302-303 states: “Together, these data indicated that Aag2 TREH is a secreted form of the enzyme and exists in the extracellular environment.” Based on what is known—that the gene explored is a homologue of TERH and the predicted results of subcellular localization—it could be suggested that the protein encoded by this gene is likely secretory. However, there is no direct evidence in this study supporting that the protein is secretory. Further investigation is needed to confirm this.

Thank you for identifying a key experiment that can reveal trehalase activity in cell-free supernatant. We were able to provide a new dataset using cell free supernatant from Aag2 cells that were grown in trehalose-containing cell culture medium to show conversion of trehalose to glucose over a 72-hour time course. Importantly, this activity was inhibited by 1 mM trehalase inhibitor validamycin A (ValA) and did not occur when trehalose-containing cell culture medium that had not contacted Aag2 cells was used. This new figure is included as Figure 1C and provides strong support that at least some TREH is secreted into the extracellular environment.

-All experiments in the study rely on trehalose or inhibitor treatment. To strengthen the findings, genetic modifications targeting specific genes, such as TREH, should also be performed to assess the direct impact of these modifications on the observed effects.

We appreciate the suggestion, but the purpose of this manuscript was to establish a mosquito cell model that relies on the major hemolymph sugar – trehalose - and use this model to characterize novel trehalase inhibitors and determine their impact on cell physiology and virus infection. The specific role of TREH and other trehalose-related proteins have been well established, and after identifying that TREH is expressed by Aag2 cells and confirming trehalase activity in these cultures, we were able to perform experiments that answered important questions about the role of trehalose and trehalase inhibitors in mosquito physiology and virus infection. A future direction that we are interested in would rely on genetic knockout/knockdown of facilitated trehalose transporter protein homologs to identify the homolog that leads to trehalose uptake in Aag2 cells and possibly peripheral tissues of live mosquitoes. We would like to know why trehalose enters into some cells when trehalase is actively secreted into the external environment *in vitro* and *in vivo*.

-If feasible, the authors should include experiments that evaluate the influence of trehalose on virus infection in mosquitoes, in order to verify the in vitro data within a more biologically relevant context.

We agree that this is an important next step. As suggested above, we are also interested in determining if trehalose can enter into peripheral cells/tissues, and if so, how does that relate to mosquito physiology and virus infection. Characterizing these relationships and molecular tools *in vitro* is an important step toward the ultimate goal of performing these experiments *in vivo*. We previously showed that ValA delays development and prevents flight in live mosquitoes, so we are ready and able to translate these *in vitro* findings in our next project.

Validamycin A Delays Development and Prevents Flight in Aedes aegypti (Diptera: Culicidae). Marten AD, Stothard AI, Kalera K, Swarts BM, Conway MJ. J Med Entomol. 2020 Jul 4;57(4):1096-1103. doi: 10.1093/jme/tjaa004.

-In Fig. 2B&E, the annotations of different shapes should be included directly in the figure, rather than in the figure legends, to improve clarity and accessibility.

Thank you. We have made that change in the revised manuscript.

-Lines 345-348 mention that differentially regulated genes are enriched in cell membrane processes, lipid biosynthesis, etc. However, the annotations in Fig. 3C&D are inconsistent with the description provided in the text. The figure should be revised to accurately reflect the described enrichments.

Thank you. The mismatch was due to a previous DAVID Functional Annotation result that was not included in the figure. We aligned the language to the g:Cost Functional Profiling tool summary, which is shown in the pie charts in Figures 3C-D. The language in the text now states, “Functional annotations of differentially expressed genes also showed significant changes in genes involved in cell-substrate adhesion, integral/intrinsic membrane components, metabolic pathways, drug metabolism, transmembrane receptor protein kinase activity, and enzyme-linked receptor protein signaling (Figure 3C-D).” We also restated that the g:Cost tool was used in the figure legend.

Reviewer #3: This study examined the role of trehalose in Aedes cell growth and dengue virus replication.

-The study is interesting, but one concern is that many of the observed phenotypes could be linked to differences in the build up of waste produced (specifically as growth differences are noted much later).

Thank you. We appreciate the comment. I think it is important to note that in Figure 2B we showed that there is no difference in growth in Aag2 cells fed glucose vs trehalose-containing cell culture media. Please see below. The differences that we see are only apparent when the trehalose-fed cells are treated with a trehalase inhibitor or infected with DENV. We also reported statistically significant changes to the transcriptome.

1. There is a wide literature base for the role of trehalose in relation to the trehalose metabolism, including knockdown studies in mosquitoes and fruit flies (see <https://doi.org/10.1038/s41598-018-24893-z>, <https://www.nature.com/articles/s42003-020-0889-1>, <https://www.mdpi.com/2075-4450/13/11/1070>). This includes studies directly on using trehalase inhibitors in control. The authors discuss some studies, but this is a much more vast field that needs coverage of more closely related studies.

We agree that there is a large literature base related to trehalose metabolism in arthropods and other species. We include 20 references in the manuscript that cover trehalase inhibitors, RNAi, and genetic knockout in different species, and a few review articles that are even more heavily cited. We have added the three additional examples provided by the reviewer.

Conway MJ, Haslitt DP, Swarts BM. Targeting Aedes aegypti Metabolism with Next-Generation Insecticides. Viruses. 2023;15(2).

Zhang L, Qiu LY, Yang HL, Wang HJ, Zhou M, Wang SG, et al. Study on the Effect of Wing Bud Chitin Metabolism and Its Developmental Network Genes in the Brown Planthopper, Nilaparvata lugens, by Knockdown of TRE Gene. Front Physiol. 2017;8:750.

Xia Y, Clarkson JM, Charnley AK. Trehalose-hydrolysing enzymes of Metarhizium anisopliae and their role in pathogenesis of the tobacco hornworm, Manduca sexta. J Invertebr Pathol. 2002;80(3):139-47.

Wyatt GR, Kale GF. The chemistry of insect hemolymph. II. Trehalose and other carbohydrates. J Gen Physiol. 1957;40(6):833-47.

Wegener G, Macho C, Schloder P, Kamp G, Ando O. Long-term effects of the trehalase inhibitor trehazolin on trehalase activity in locust flight muscle. J Exp Biol. 2010;213(Pt 22):3852-7.

Wegener G, Tschiedel V, Schloder P, Ando O. The toxic and lethal effects of the trehalase inhibitor trehazolin in locusts are caused by hypoglycaemia. J Exp Biol. 2003;206(Pt 7):1233-40.

Thorat LJ, Gaikwad SM, Nath BB. Trehalose as an indicator of desiccation stress in Drosophila melanogaster larvae: a potential marker of anhydrobiosis. Biochem Biophys Res Commun. 2012;419(4):638-42.

Tatun N, Wangsantitham O, Tungjitwitayakul J, Sakurai S. Trehalase activity in fungus-growing termite, Odontotermes feae (Isoptera: Termitideae) and inhibitory effect of validamycin. J Econ Entomol. 2014;107(3):1224-32.

Tang B, Yang M, Shen Q, Xu Y, Wang H, Wang S. Suppressing the activity of trehalase with validamycin disrupts the trehalose and chitin biosynthesis pathways in the rice brown planthopper, Nilaparvata lugens. Pestic Biochem Physiol. 2017;137:81-90.

Shukla E, Thorat LJ, Nath BB, Gaikwad SM. Insect trehalase: physiological significance and potential applications. Glycobiology. 2015;25(4):357-67.

Marten AD, Stothard AI, Kalera K, Swarts BM, Conway MJ. Validamycin A Delays Development and Prevents Flight in Aedes aegypti (Diptera: Culicidae). J Med Entomol. 2020;57(4):1096-103.

Katagiri N, Ando O, Yamashita O. Reduction of glycogen in eggs of the silkworm, Bombyx mori, by use of a trehalase inhibitor, trehazolin, and diapause induction in glycogen-reduced eggs. J Insect Physiol. 1998;44(12):1205-12.

Liebl M, Nelius V, Kamp G, Ando O, Wegener G. Fate and effects of the trehalase inhibitor trehazolin in the migratory locust (Locusta migratoria). J Insect Physiol. 2010;56(6):567-74.

Chen J, Zhang D, Yao Q, Zhang J, Dong X, Tian H, et al. Feeding-based RNA interference of a trehalose phosphate synthase gene in the brown planthopper, Nilaparvata lugens. Insect Mol Biol. 2010;19(6):777-86.

Chen J, Tang B, Chen H, Yao Q, Huang X, Zhang D, et al. Different functions of the insect soluble and membrane-bound trehalase genes in chitin biosynthesis revealed by RNA interference. PLoS One. 2010;5(4):e10133.

Asano N, Yamaguchi T, Kameda Y, Matsui K. Effect of validamycins on glycohydrolases of Rhizoctonia solani. J Antibiot (Tokyo). 1987;40(4):526-32.

Murphy TA, Wyatt GR. ENZYMATIC REGULATION OF TREHALOSE AND GLYCOGEN SYNTHESIS IN THE FAT BODY OF AN INSECT. Nature. 1964;202:1112-3.

Becker A, Schloder P, Steele JE, Wegener G. The regulation of trehalose metabolism in insects. Experientia. 1996;52(5):433-9.

Arguelles JC. Why can't vertebrates synthesize trehalose? J Mol Evol. 2014;79(3-4):111-6.

Yuan S, Zhang ZW, Li ZL. Trehalose May Decrease the Transmission of Zika Virus to the Fetus by Activating Degradative Autophagy. Front Cell Infect Microbiol. 2017;7:402.

Wu Q, Jiang D, Huang C, van Dyk LF, Li L, Chu HW. Trehalose-mediated autophagy impairs the anti-viral function of human primary airway epithelial cells. PLoS One. 2015;10(4):e0124524.

2. Line 81-88 – This entire paragraph has little importance and should be deleted.

Thank you. We are interested in the use of trehalase inhibitors due to their safety profile in humans, but we agree that this paragraph can be removed from the revised manuscript.

3. Most of the differences in cell growth seem to occur after day 5. Is it possible that trehalose is metabolized slower and can be used over longer periods? A steady nutrient release over extended periods could promote more growth

Thank you. We appreciate the comment. I think it is important to note that in Figure 2B we showed that there is no difference in growth in Aag2 cells fed glucose vs trehalose-containing cell culture media. Please see below. The differences that we see are only apparent when the trehalose-fed cells are treated with a trehalase inhibitor or infected with DENV. We also reported statistically significant changes to the transcriptome.

4. Statistical description as inadequate. There is no discussion of normality, etc. Also, there is just a general lack of description of how the data was analyzed. T-tests are likely not the most appropriate test in some cases.

Thank you. We have described our statistical analysis in more detail in each figure legend and indicated when results were non-significant in the figure. Here is an example of a revised figure legend statement, “Assays were performed in triplicate. Normality and unpaired Student’s t tests were performed between groups to assess statistical significance (asterisks indicate statistical significance, * p < 0.01, ** p<0.001, *** p<0.0001, ns indicates non-significance). Standard deviations are shown.”

5. Results – line 298: The description does not match the figure

Thank you. This section did need to be clarified after review. We have found that there is expression of TREH in Aag2 cells, but that the TPS (AAEL006446) gene that was automatically predicted from the *Ae. aegypti* genome sequence was absent in the analysis. TPP (AAEL010684) was predicted by UnitProtKB/TrEMBL and is reported in VectorBase but is low homology when compared with TPP from other species. We originally placed a question mark because we were skeptical that this was the real TPP, although we don’t have evidence to support that one way or the other. The question mark has been removed. We revised the language in the manuscript to reflect this. “An RNA-Seq dataset that characterized gene expression of Aag2 cells revealed high expression of trehalase (TREH; AAEL009658), but the previously predicted trehalose-6-phosphate synthase (TPS; AAEL006446) was absent in the analysis (42). The gene that provides trehalose 6-phosphate phosphatase (TPP; AAEL010684) activity in *Ae. aegypti* was predicted by UniProtKB/TrEMBL and had high expression in Aag2 cells (Figure 1A). Based on this information, it was unclear if Aag2 cells could synthesize trehalose, but they could likely metabolize trehalose.”

We added more information in the figure legend so that the reader can identify the exact location of the predicted signal peptide and understand that the plots identify the n-terminal, hydrophobic, and c-terminal regions of the signal peptide. The new language is as follows, “(B) SignalP 6.0 prediction of a Sec/SPI signal peptide at S30 and associated n-terminal (n), hydrophobic (h), and c-terminal (c) regions in TREH; AAEL009658.”

6. Figure 2C and D. AS cell growth increased, I’d expect there would be a decline in glucose.

This was also our expectation, but after experimentation it turns out that high glucose DMEM with 450 mg/dL glucose contains more glucose than Aag2 cells need for growth. We were able to use no glucose DMEM and supplement with 10% FBS and 1% tryptose phosphate, which contains ~100 mg/dL glucose, and get comparable cell proliferation as with high glucose DMEM. The data show that 20% of the glucose is metabolized when high glucose DMEM is used over a 3 day time course. There is a lot of glucose remaining in the media when cells become confluent and it is time to split the cell culture.

7. Line 335 – this is the circulating blood sugar and it is expected that enzymes involved in trehalose expression are expressed in many tissues.

Thank you. We understand that cells outside of the fat body may express trehalase, trehalose transporters, and trehalose synthetic machinery, but this has not been described in detail. There is a gap in our understanding about how trehalose interacts with cells outside of the fat body as a functional molecule rather than a source of glucose.

8. Line 471 – As trehalose was the only factor that could serve as a substantial carbon source, the cells could be moving to a more quiescent state. As such, viral production may be reduced as Aag2 cells are no longer growing and replicating. These studies should likely be conducted in the presence of glucose (maybe 25%), which may confirm if this is due to a lack of general cell metabolism or only suppression of trehalose breakdown.

Thank you. This is a very interesting direction. As we stated above, the 10% FBS and 1% tryptose phosphate provide ~100 mg/dL glucose and free fatty acids (FFAs). These would also be used by trehalose-fed cells, so trehalose isn’t the only carbon source. We confirmed this by performing additional studies of cell proliferation in the absence of supplemental glucose, and we found that Aag2 cells grew similarly with and without the excess glucose found in high glucose DMEM.

We also performed identical cell viability, DENV shedding, and DENV infectivity assays at 100%, 50%, 10%, and 0% glucose as were performed with the glucose and trehalose-fed cells. Reducing the excess glucose concentration down to 0% had no impact on either of these variables. This suggests that ValA and trehalose have an interaction that leads to cell toxicity that we currently do not understand. It is likely not due to a hypoglycemic state. Our current hypothesis is that ValA restricts the breakdown of trehalose and excess trehalose is toxic to cells. Trehalase may help cells survive in the presence of excess trehalose. These data have been included as a new Figure 10.

9. The discussion is heavily repetitive to the results and needs to be edited to provide more

Thank you. The discussion follows the results point-by-point and expands on each finding with available literature. We would be happy to provide more information if there is a gap that we missed.

10. Sample sizes and replication are not clear in most sections.

Thank you. In each figure legend it states that assays were performed in triplicate.

11. For all box plot, I would suggest overlay of the specific points onto the plots.

Thank you. We experimented with different presentations of the data. For the simpler bar graphs, it is possible to overlay the specific points onto the plots without creating too much clutter.

The few larger and more complex bar graphs can’t accommodate layering each datapoint at their current sizes.

We have added layers to most of the figures.

12. References are not uniform

Thank you. These will be formatted to PLoS Pathogen’s requirements before publishing.

Reviewer #3: 8. Line 471 – As trehalose was the only factor that could serve as a substantial carbon source, the cells could be moving to a more quiescent state. As such, viral production may be reduced as Aag2 cells are no longer growing and replicating. These studies should likely be conducted in the presence of glucose (maybe 25%), which may confirm if this is due to a lack of general cell metabolism or only suppression of trehalose breakdown.

Thank you. This is a very interesting direction. As we stated above, the 10% FBS and 1% tryptose phosphate provide ~100 mg/dL glucose and free fatty acids (FFAs). These would also be used by trehalose-fed cells, so trehalose isn’t the only carbon source. We confirmed this by performing additional studies of cell proliferation in the absence of supplemental glucose, and we found that Aag2 cells grew similarly with and without the excess glucose found in high glucose DMEM.

We also performed identical cell viability, DENV shedding, and DENV infectivity assays at 100%, 50%, 10%, and 0% glucose as were performed with the glucose and trehalose-fed cells. Reducing the excess glucose concentration down to 0% had no impact on either of these variables. This suggests that ValA and trehalose have an interaction that leads to cell toxicity that we currently do not understand. It is likely not due to a hypoglycemic state. Our current hypothesis is that ValA restricts the breakdown of trehalose and excess trehalose is toxic to cells. Trehalase may help cells survive in the presence of excess trehalose. These data have been included as a new Figure 10.
